# Integrated Organic–Inorganic Fertilization Enhances Microbial Stoichiometric Homeostasis but Triggers Seasonal Metabolic Trade-Offs in an Alpine Sandy Ecosystem

**DOI:** 10.3390/microorganisms14061186

**Published:** 2026-05-25

**Authors:** Kai Yang, Fuchun Huang, Wensheng Yang, Xupeng Lu, Zhengtao Zhu, Jianqiang Zhu, Qixia Wu, Xiaohong Xu

**Affiliations:** 1 College of Agriculture, Yangtze University, Jingzhou 434025, China; yangkai990216@163.com (K.Y.); 2023710807@yangtzeu.edu.cn (F.H.); 13720177791@163.com (W.Y.); 2023720931@yangtzeu.edu.cn (X.L.); 13264691805@163.com (Z.Z.); qixiawu@yangtzeu.edu.cn (Q.W.); 2 College of Earth Sciences, Yangtze University, Wuhan 430100, China; 18993603436@163.com

**Keywords:** elemental use efficiency, walnut plantations, soil chemistry stoichiometry, rhizosphere and non-rhizosphere, seasonal variation, fertilization management

## Abstract

The ecological restoration of degraded sandy land in the Yarlung Zangbo River Valley is constrained by the metabolic functions of soil microorganisms. This study investigates the dynamic mechanisms of microbial elemental use efficiency in walnut plantations, with a focus on seasonal variations in soil chemical stoichiometry, extracellular enzyme activity, and microbial nutrient efficiency in rhizosphere and bulk soils. This paper explores the effects of conventional organic fertilizer (CF) and organic–inorganic compound fertilizer (OIF) on microbial nutrient use strategies and their seasonal dynamics. The results showed significant seasonal fluctuations in soil active nutrients and microbial biomass, while the total nutrient content remained stable. OIF enhanced microbial chemical stoichiometric homeostasis but simultaneously triggered a “carbon–phosphorus metabolic trade-off”, leading to a restraint of microbial carbon use efficiency (CUE) during the growing season. Microbial elemental use efficiency (EUE) exhibited clear seasonal differentiation: CUE was higher in summer, promoting biomass accumulation, whereas NUE and PUE increased in winter and spring, reflecting a nutrient conservation strategy. The EUE pathways were decoupled between rhizosphere and non-rhizosphere microenvironments. The rhizosphere was more directly driven by soil chemical stoichiometry and microbial biomass, while the non-rhizosphere was influenced by nutrient limitation states, represented by vector characteristics. This study provides insights into the seasonal adaptability and microenvironmental heterogeneity of microbial metabolism during the restoration of cold sandy land. It is suggested that future ecological management should focus on N-P balanced fertilization and consider the differential responses between rhizosphere and non-rhizosphere zones to enhance ecosystem productivity and soil carbon, nitrogen, and phosphorus sequestration potential.

## 1. Introduction

As critical components of the global terrestrial biosphere, alpine and dryland ecosystems are essential for carbon sequestration and climate stability, yet they are increasingly vulnerable to degradation under the pressures of global change [1]. The Tibetan Plateau, known as the “Water Tower of Asia” and an important biodiversity hotspot globally, plays a crucial role in maintaining regional climate regulation and water conservation. However, due to the dual impacts of global climate change and human activities, the desertification of plateau lands has become an increasingly severe issue, which has led to vegetation degradation, soil nutrient loss, and an imbalance in microbial functions [2,3,4]. Previous research in this region, including the establishment of artificial grasslands and N/P fertilization, has shown that while these measures can significantly enhance biomass and soil nutrients, they often fail to fully restore soil functions and microbial community structures to their original pristine state. Furthermore, N and P addition has been found to trigger complex trade-offs in forage quality and soil acidification, highlighting the need for more precise nutrient management [5,6]. To rehabilitate degraded sandy lands and improve livelihoods, the government has launched afforestation projects, including walnut (*Juglans regia*) plantations in the middle reaches of the Yarlung Zangbo River [7]. While vegetation restoration has improved soil physical properties, the harsh environmental conditions of the plateau, including extreme diurnal temperature fluctuations, frequent freeze–thaw cycles, and seasonal precipitation variability, significantly disrupt the ecological functions of soil microorganisms, limiting the long-term success of ecological restoration [8,9].

Microbial element use efficiency (EUE), which includes carbon use efficiency (CUE), nitrogen use efficiency (NUE), and phosphorus use efficiency (PUE), quantifies the ability of microorganisms to convert absorbed nutrients into biomass. It is a key indicator for assessing soil carbon, nitrogen, and phosphorus sequestration potential, as well as the stability of ecosystems. Specifically, CUE directly regulates the rate of soil organic carbon accumulation. A higher CUE means that microorganisms prioritize allocating carbon resources to growth rather than respiration, thereby enhancing carbon sequestration and mitigating climate change [10,11]. However, CUE is regulated by multiple factors, including temperature, humidity, and substrate quality, with its response mechanisms being highly environment-dependent [11,12,13]. Similarly, the dynamic changes in NUE and PUE are closely related to soil nutrient availability, enzyme activity stoichiometric balance, and the functional traits of microbial communities [14,15]. Although the theoretical framework for microbial element use efficiency has been increasingly refined, most studies have focused on the efficiency of individual elements. Furthermore, the concept of stoichiometric homeostasis is vital to understanding how microbes maintain stable internal C:N:P ratios despite external resource fluctuations. However, the linkage between homeostasis and multi-element use efficiency remains poorly understood in the alpine restoration context. There is still a lack of a systematic understanding of the synergistic responses and seasonal variation mechanisms of C-N-P use efficiency during the restoration of cold sandy land.

The rhizosphere is the most active microenvironment in plant–soil–microbe interactions. Plant root exudates and nutrient absorption processes modify the rhizosphere microenvironment, causing rhizosphere microorganisms to differ significantly in metabolic activity and functional traits from those in bulk soil. This microenvironmental heterogeneity often leads to a “decoupling” of microbial metabolic pathways. Specifically, while labile carbon input from roots may alleviate microbial carbon limitation in the rhizosphere, it simultaneously intensifies competition for nitrogen and phosphorus between plants and microbes, forcing a shift in resource allocation toward specific nutrient-acquiring enzymes [16]. To quantify these shifts, vector analysis of extracellular enzymes provides a robust framework, where vector length and angle represent the magnitude and type of nutrient limitation, respectively [17]. Fertilization, as a key measure in the management of artificial forests, directly supplements available nutrients and alleviates microbial resource limitations, thereby reshaping the stoichiometric balance of C:N:P in the soil [18]. In cold environments, how the interaction between fertilization and rhizosphere effects regulates the physiological homeostasis of microorganisms and their EUE trade-off strategies is crucial for evaluating the ecological restoration of artificial forests. For instance, fertilization might enhance CUE by alleviating carbon limitation, but it could also intensify microbial competition for phosphorus, forcing microorganisms to invest more energy in synthesizing phosphatases, thus leading to a shift in the metabolic trade-off between CUE and PUE [19,20].

Moreover, the strong seasonal variation in cold regions (e.g., spring freeze–thaw cycles, summer water–heat synchronization, and winter low temperatures) constitutes stress factors that drive complex periodic fluctuations in microbial metabolism and EUE. However, there is significant debate regarding the response patterns of microbial EUE to seasonal environmental changes. On the one hand, soil temperature fluctuations and moisture stress may increase osmotic regulation costs and suppress respiration, thereby inhibiting CUE [21,22,23]. On the other hand, some studies suggest that microorganisms can maintain a relatively stable EUE through physiological compensation mechanisms [24,25]. Similarly, the responses of NUE and PUE to temperature and moisture stress are also inconsistent. Some studies suggest that NUE responds positively to temperature increases with minimal impact from moisture changes [26], while others indicate that extreme climates significantly affect both NUE and CUE [14]. This uncertainty is particularly evident in cold sandy land restoration areas, as management measures, such as walnut planting and fertilization, interact strongly with natural seasonal fluctuations, thereby regulating EUE [16,27]. For example, under low winter and spring temperatures, nitrogen fertilization may alleviate carbon limitation [28], but in the context of seasonal drought, it often intensifies phosphorus competition between plants and microorganisms, triggering a “carbon–phosphorus metabolic trade-off” and leading to a significant decrease in CUE [17,18]. This creates a critical ecological paradox: while fertilization is intended to alleviate nutrient limitations, it may inadvertently force microorganisms to divert carbon from growth toward costly enzyme synthesis, thereby undermining soil carbon sequestration. However, how fertilization-induced nutrient limitations reshape the C-N-P use efficiency in rhizosphere and non-rhizosphere microenvironments across seasons remains a critical issue to address. Investigating these dynamics provides not only precise management strategies for local walnut plantations but also a broader template for understanding microbial resilience during the ecological restoration of degraded alpine lands worldwide.

Therefore, this study focuses on walnut plantations in the Yarlung Zangbo River Valley of the Tibetan Plateau. We established an experimental system involving “conventional organic fertilization (CF) and combined organic and inorganic fertilization (OIF).” Through seasonal soil sampling of rhizosphere and bulk soils (March, July, and November), combined with extracellular enzyme stoichiometry, vector analysis, and partial least squares path modeling (PLS-PM), this study aimed to test the following core hypotheses: (1) the rhizosphere effect and fertilization optimize soil C:N:P balance, enhancing microbial CUE and improving microbial stoichiometric homeostasis; (2) microbial EUE exhibits significant seasonal variation, with high CUE in summer (July) promoting biomass accumulation, while spring and winter (March and November) increase NUE/PUE to cope with nutrient scarcity; and (3) fertilization reshapes the regulatory pathways of EUE in rhizosphere and bulk soils by altering extracellular enzyme activity and nutrient limitation states. The results of this study will reveal the dynamic characteristics of soil–microbe metabolic coupling in the restoration of cold sandy land, providing scientific guidance for precise fertilization management and ecological function evaluation of artificial forests.

## 2. Materials and Methods

### 2.1. Experimental Site Description

The experimental site is located in Jiacha County, Shannan City, the Tibet Autonomous Region, within the broad valley area of the middle and lower reaches of the Yarlung Zangbo River, where the elevation ranges from 3000 to 3300 m, and its climate is a temperate plateau semi-arid monsoon climate with significant temperature fluctuations. The area has an average annual temperature of 9.4 °C, and the average annual precipitation is 492 mm, which is mainly concentrated from May to September, while the average annual evaporation ranges from 2000 to 2200 mm. The field experiments were made at the Tibet Plateau Walnut Industry Research Institute of Yangtze University (29°4′29″ N, 92°44′41″ E). Prior to the experiment, the soil was classified as degraded sandy land, with vegetation predominantly consisting of drought-tolerant and nutrient-poor species, such as *Neotrinia splendens*, *Artemisia gmelinii*, and *Erodium cicutarium*. The parent material of the soil consists mainly of river alluvial deposits, and the soil types include subalpine meadow soils and gray–brown soils, with textures primarily composed of sandy loam and sandy soils, and a high sand content.

### 2.2. Experimental Design

In March 2022, three-year-old walnut seedlings with uniform growth were planted in the degraded sandy plots with a row spacing of 5 m and a plant spacing of 4 m. The experiment included two fertilization treatments: (1) local conventional fertilization (CF) and (2) combined application of organic and inorganic fertilizers (OIFs). Each treatment was established with six replicate plots, giving a total of twelve experimental plots, each with an area of 0.1 hm^2^. Under the CF treatment, 30 kg of well-decomposed cattle manure was applied per tree as basal fertilizer at planting (OM = 14.5%, N = 0.35%, P_2_O_5_ = 0.20%, and K_2_O = 0.12%), with no additional fertilization thereafter. Under the OIF treatment, the same basal application of 30 kg of well-decomposed cattle manure per tree was applied at planting, followed by topdressing with a humic acid-containing organic–inorganic water-soluble fertilizer each year in April and September (humic acid ≥ 200 g L^−1^, N ≥ 100 g L^−1^, P_2_O_5_ ≥ 100 g L^−1^, and K_2_O ≥ 60 g·L^−1^). Basal fertilizer was applied by first excavating planting pits to a depth of 40 cm, thoroughly mixing the cattle manure with the soil, and then backfilling before walnut seedlings were planted. The water-soluble fertilizer was supplied through an integrated fertigation drip-irrigation system, with a total of 360 kg applied over the 2022–2025 period.

### 2.3. Sample Collection and Analysis

#### 2.3.1. Soil Sample Collection

Soil sampling was conducted in March, July, and November 2025, during which five walnut trees with good growth performance and uniform spatial distribution were selected from each experimental plot. To ensure the robustness of the statistical analysis and the spatial representativeness of the samples, while minimizing random variation caused by microsite heterogeneity, soil samples collected from the five trees were composited into a single sample, with six replicates per treatment (*n* = 6). To ensure consistency in soil nutrient distribution among samples, soil was collected from multiple points within the drip-irrigated zone at a distance of 50–80 cm from the trunk and at a depth of 5–20 cm. After removing surface debris, the fine roots were gently excavated, and the adhering soil was carefully collected and put into sterile zip-lock bags as rhizosphere soil, while samples taken from areas away from the roots were designated as bulk soil. To minimize within-plot variability, samples from ten trees per plot were combined into a single composite sample. The samples were cleaned of plant residues and gravel and then divided into two subsamples. One of them was sieved through a 2 mm mesh and stored at −20 °C for microbial biomass analysis, while the other was air-dried and sieved for analysis of soil chemical properties and enzyme activity and stored at 4 °C.

#### 2.3.2. Soil Chemical Properties and Extracellular Enzyme Activity Analysis

After air drying and sieving (1 mm and 0.149 mm), soil properties were determined according to Bao’s [29] protocols. Soil pH was measured using a glass electrode with a soil-to-water ratio of 2.5:1. Organic matter (OM) was determined by K_2_Cr_2_O_7_ oxidation and external heating. The detection method for total nitrogen (TN) was digestion with H_2_SO_4_, followed by flow injection analysis; for total phosphorus (TP), it was NaOH fusion using the molybdenum–antimony colorimetry method. Microbial biomass (MBC, MBN, and MBP) was determined using the chloroform fumigation method [30]. Soluble organic carbon (DOC) and soluble nitrogen (DN) were extracted using 0.5 mol/L of K_2_SO_4_ and determined by a TOC analyzer, while soluble phosphorus (DP) was extracted with 0.5 mol/L of NaHCO_3_ and determined by the molybdenum–antimony colorimetric method [27]. Soil enzymes, including β-glucosidase (BG), leucine aminopeptidase (LAP), N-acetyl-β-D-glucosaminidase (NAG), and alkaline phosphatase (ALP), were analyzed following standard procedures provided by enzyme reagent kits (Suzhou Grace Biotechnology Co., Ltd., Suzhou, China).

#### 2.3.3. Soil Microbial Nutrient Limitation Analysis

The nutrient limitation of microbial communities was characterized by the stoichiometric imbalance between available soluble nutrients and microbial biomass. The following formulas were used for calculation [31]:

(1) $$C:N_{imbalance}=\frac{\left(DOC:DN\right)}{\left(MBC:MBN\right)}$$

(2) $$C:P_{imbalance}=\frac{\left(DOC:DP\right)}{\left(MBC:MBP\right)}$$

(3) $$N:P_{imbalance}=\frac{\left(DN:DP\right)}{\left(MBN:MBP\right)}$$

To assess seasonal resource limitations, the vector characteristics of extracellular enzymes were calculated using the following formula [32]:

(4) $$VL=\sqrt{X^{2}+Y^{2}}$$

(5) VA=DEGREES (ATAN2(X,Y))

where *X* represents BG/(BG + ALP), *Y* represents BG/(BG + NAG + LAP), *VL* represents the vector length of extracellular enzymes (higher *VL* values indicate stronger C-limitation), and *VA* represents the vector angle (indicating the degree of N and P limitation). *VA* < 45° indicates N limitation, with smaller angles indicating stronger N limitation, while *VA* > 45° indicates P limitation, with larger angles indicating stronger P limitation.

Microbial chemical stoichiometric stability was calculated as follows [33]:

(6) $$H^{\prime }=1/m$$

where *m* represents the regression slope between *ln*(*SOC*:*TN*) and *ln*(*MBC*:*MBN*) or *ln*(*SOC*:*TP*) and *ln*(*MBC*:*MBP*). This slope indicates the stability of soil microbial elements, where *H’* > 1 represents strong stability, and H’ ≈ 1 represents weak stability.

#### 2.3.4. Analysis of Soil Microbial Element Use Efficiency

Carbon use efficiency (CUE) was calculated as follows [32]:

(7) $$CUE=CUE_{max}{\left[\frac{S_{C:N}\times S_{C:P}}{\left(S_{C:N}+K_{x})\times (S_{C:P}+K_{x}\right)}\right]}^{0.5}$$

(8) $$S_{CX}=\frac{1}{EEA_{C:X}}\times \frac{B_{C:X}}{L_{C:X}}$$

where *CUE_max_* = 0.6 represents the thermodynamic upper limit of microbial carbon use efficiency. *K_x_* = 0.5 is the half-saturation coefficient. *S_C:X_* represents the degree to which extracellular enzyme activity compensates for the differences between the elemental composition of available resources and microbial biomass composition. *EEA_C_*_:*X*_ represents *BG/(LAP + NAG)* or *BG/AKP*, and *B_C_*_:*X*_ represents *MBC*:*MBN* or *MBC*:*MBP*, with *L_C_*_:*X*_ representing *SOC*:*TN* or *SOC*:*TP*.

Nitrogen (NUE) and phosphorus (PUE) use efficiency were calculated as follows:

(9) $$XUE=XUE_{max}\times S_{X:C}\times S:C+K_{C}$$

(10) $$S_{\overset{-}{X}C}=\frac{1}{EEA_{X:C}}\times \frac{B_{X:C}}{L_{X:C}}$$

where *XUE* refers to *NUE* or *PUE*, *XUE_max_* = 1.0 represents the thermodynamic upper limit of microbial nitrogen and phosphorus use efficiency, *K_C_* = 0.5 is the half-saturation coefficient, *EEA_X_*_:*C*_ represents *(LAP + NAG):BG* or *ALP*:*BG*, *B_X_*_:*C*_ represents *MBN*:*MBC* or *MBP:MBC*, and *L_X_*_:*C*_ represents *TN*:*SOC* or *TP*:*SOC*.

### 2.4. Statistical Analysis

Data were analyzed using the DPS 9.50 software for two-way ANOVA to examine significant seasonal differences in soil chemical properties, extracellular enzyme activity, elemental vector characteristics, and element use efficiency. Spearman correlation analysis was used to explore the relationships between soil chemical and biological properties. Mantel tests were performed to calculate the correlation between extracellular enzyme vector characteristics and soil biochemical properties. Statistical analysis and plotting were performed using the ChiPlot platform (https://www.chiplot.online/ accessed on 2 January 2026). PLS-PM was constructed using the SmartPLS 4 software to investigate the impact of soil stoichiometry, microbial biomass, extracellular enzyme stoichiometry, and vector characteristics on element use efficiency. Bar charts, scatter plots, and linear fitting analysis were generated using the Origin 2021 software. All results are presented as means ± standard error (SE).

## 3. Results

### 3.1. Seasonal Dynamics of Total and Labile Nutrient Pools Under Fertilization and Rhizosphere Regulation

The stoichiometric indicators exhibited varying degrees of seasonal variation across treatments (Table 1). Overall, soil TN and TP tended to decrease first and then increase across seasons, although no significant seasonal differences were detected. Under the CF treatment, TN in both rhizosphere (*p* < 0.01) and bulk soils (*p* < 0.05) was significantly lower in July than in March. In contrast, TN in both rhizosphere and bulk soils under OIF showed no significant seasonal change. TP did not display significant seasonal dynamics in any treatment. DOC in both rhizosphere (*p* < 0.05) and bulk (*p* < 0.01) soils under CF was significantly lower in July than in March. Similarly, DOC in rhizosphere soil under OIF was significantly lower in July than in March (*p* < 0.05). In contrast, DN and DP showed no significant variation in either rhizosphere or bulk soils under CF. Under the OIF, significant changes in DN and DP were observed only in the rhizosphere soil, where both were significantly lower in July than in November (*p* < 0.01). MBC under CF showed a continuous decline in both rhizosphere and bulk soils and was significantly lower in November than in March (*p* < 0.05). In contrast, MBC under CF decreased first and then increased in both soil compartments, with values in July significantly lower than those in March and November. MBN under OIF followed a similar pattern, with significantly lower values in July than in March (*p* < 0.05) and November (*p* < 0.01). MBP generally decreased first and then increased across treatments, and MBP in bulk soil under OIF was significantly lower in July than in November (*p* < 0.05).

### 3.2. Seasonal Responses of C, N, and P Acquiring Enzyme Activities and Their Fertilization-Dependent Differences

The extracellular enzyme activities showed pronounced seasonal variation (Table 2). The activities of BG, LAP, NAG, and ALP all increased first and then declined across seasons. Under CF, BG activity in both rhizosphere (*p* < 0.01) and bulk (*p* < 0.05) soils was significantly higher in July than in March. LAP activity in both rhizosphere and bulk soils under both CF (*p* < 0.01) and OIF (*p* < 0.05) was significantly higher in July than in March. Moreover, under CF, LAP activity in both rhizosphere (*p* < 0.01) and bulk (*p* < 0.05) soils was also significantly higher in July than in November. Under CF, NAG activity in rhizosphere soil was significantly higher in July than in March (*p* < 0.01) and November (*p* < 0.05), whereas no significant seasonal change was observed in bulk soil. Under OIF, NAG activity in both rhizosphere and bulk soils was significantly higher in July than in March (*p* < 0.01). ALP activity under CF in both soil compartments was significantly higher in July than in March and November (*p* < 0.01). Similarly, rhizosphere ALP activity under OIF was significantly higher in July than in March and November (*p* < 0.01), while no significant seasonal differences were detected in bulk soil.

### 3.3. Seasonal Evolution of Microbial Nutrient Limitation Inferred from Enzyme Vectors and Stoichiometric Imbalance Indices

**Figure 1:**
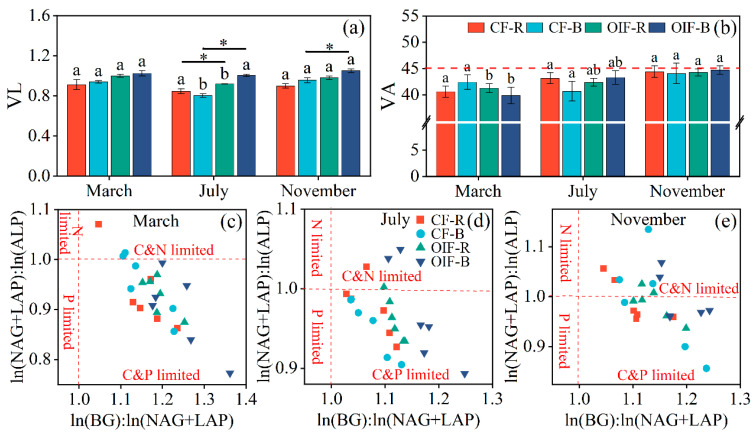
Extracellular enzyme vector characteristics under different treatments. (**a**) Vector length of extracellular enzymes, (**b**) vector angle of extracellular enzymes, and (**c**–**e**) nutrient limitations on microbial metabolism under each treatment in March, July, and November. Ln(BG), ln(NAG + LAP), and ln(ALP) represent the log-transformed values of BG, NAG + LAP, and ALP, respectively. Different lowercase letters indicate significant differences between the same treatment in different months (*p* < 0.05), and * indicates significant differences between different treatments in the same month (*p* < 0.05).

The enzyme vector model revealed clear seasonal patterns (Figure 1). Across the temporal scale, vector length (VL) under CF was significantly higher in March and November than in July (*p* < 0.05) (Figure 1a). In July, VL values in both rhizosphere and bulk soils under OIF were significantly higher than those under CF (*p* < 0.05). In November, VL in bulk soil under OIF also significantly exceeded that under CF (*p* < 0.05) (Figure 1a). Notably, vector angles (VAs) were consistently <45° across all treatments (Figure 1b), indicating that microbial metabolism was jointly constrained by both C-N and C-P limitations. This pattern was consistent with the distribution of extracellular enzyme stoichiometric ratios (Figure 1c–e).

**Figure 2:**
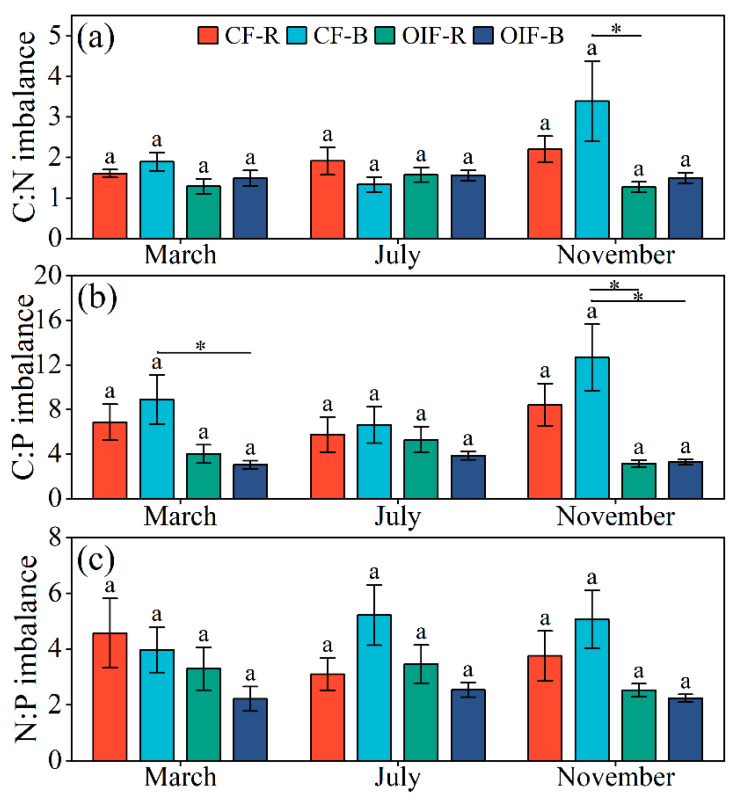
Nutrient element imbalance characteristics under different treatments. (**a**) C:N imbalance: C-N stoichiometric imbalance; (**b**) C:P imbalance: C-P stoichiometric imbalance; (**c**) N:P imbalance: N-P stoichiometric imbalance. Different lowercase letters indicate significant differences between the same treatment in different months (*p* < 0.05), and * indicates significant differences between different treatments in the same month (*p* < 0.05).

Further analysis showed that, in both rhizosphere and bulk soils, the degree of C:P imbalance was greater than that of C:N and N:P imbalance (Figure 2). C:N imbalance was most evident in November, particularly in both soil compartments under CF (Figure 2a). In March and November, C:P imbalance in bulk soil under CF was significantly higher than that under OIF (*p* < 0.05) (Figure 2b). Overall, C:N, C:P, and N:P imbalance indices tended to be higher under CF than under OIF (Figure 2a–c), although differences among treatments and seasons were not statistically significant.

### 3.4. Seasonal Differentiation of Microbial C, N, and P Use Efficiency and Shifts in Metabolic Strategies

**Figure 3:**
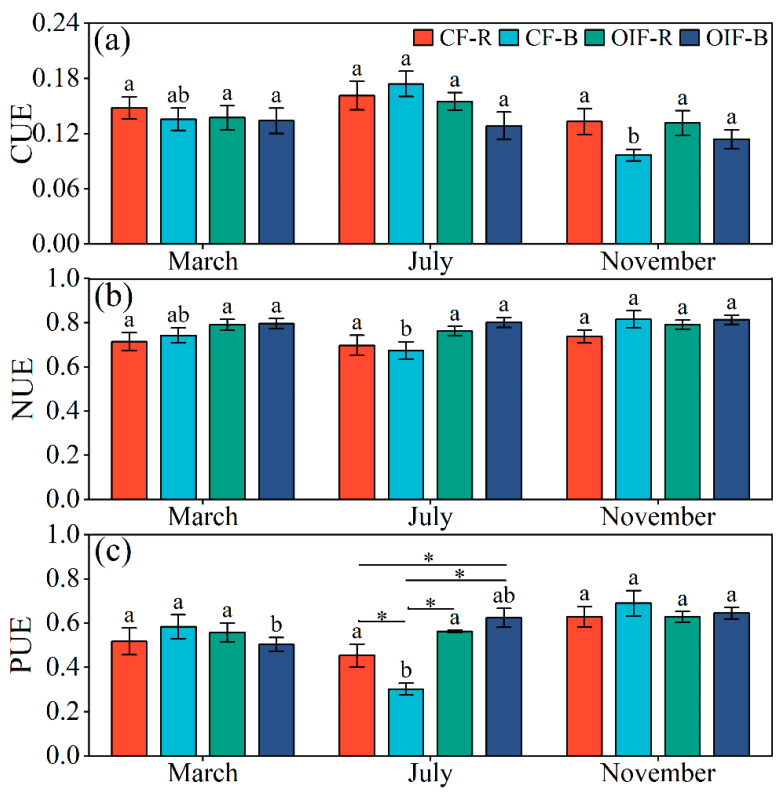
Microbial element use efficiency under different treatments. (**a**) CUE: carbon use efficiency; (**b**) NUE: nitrogen use efficiency; (**c**) PUE: phosphorus use efficiency. Different lowercase letters indicate significant differences between the same treatment in different months (*p* < 0.05), and * indicates significant differences between different treatments in the same month (*p* < 0.05).

CUE in both rhizosphere and bulk soils peaked in July, followed by March, and reached the lowest values in November (Figure 3a). Specifically, CUE in bulk soil under CF was significantly higher in July than in November (*p* < 0.05) (Figure 3a). In contrast, NUE was generally higher in November and lower in July, and in bulk soil under CF, it was significantly higher in November than in July (*p* < 0.05) (Figure 3b). PUE also tended to be higher in November and lower in July, and in bulk soil under CF, it was significantly higher in November than in July, whereas PUE in rhizosphere soil under OIF was significantly higher in November than in March (*p* < 0.05) (Figure 3c). Notably, PUE displayed relatively large treatment-dependent differences in July. Significant differences were detected between rhizosphere and bulk soils under CF, as well as between OIF and CF in bulk soils.

### 3.5. Responses of Microbial Biomass Stoichiometric Homeostasis to Fertilization Management

**Figure 4:**
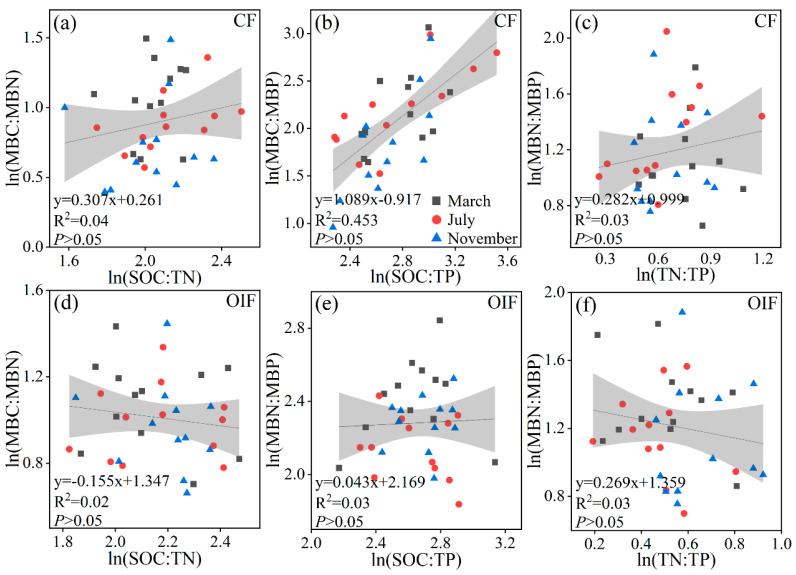
Relationships between log-transformed soil microbial biomass and total nutrient stoichiometric ratios under different treatments. (**a**–**c**) Relationships between microbial biomass C:N, C:P, and N:P and soil total C:N, C:P, and N:P under CF treatment; (**d**–**f**) relationships between microbial biomass C:N, C:P, and N:P and soil total C:N, C:P, and N:P under OIF treatment. The inverse of the equation slope represents the stability of microbial elements, with larger values indicating stronger microbial homeostasis. Gray shading represents the 95% confidence interval.

Regression analyses between microbial biomass C:N:P ratios and corresponding soil substrate C:N:P ratios indicated that, under CF, the slopes of ln(MBC:MBN) vs. ln(SOC:TN), ln(MBC:MBP) vs. ln(SOC:TP), and ln(MBN:MBP) vs. ln(TN:TP) were 0.307, 1.089, and 0.082, respectively (Figure 4a–c). The corresponding H’ values were 3.3, 0.9, and 3.5, indicating relatively weak stoichiometric homeostasis. Under OIF, the corresponding slopes were 0.155, 0.043, and 0.269 (Figure 4d–f), with H’ values of 6.5, 23.3, and 3.7. These results suggest stronger stoichiometric homeostasis, although the correlations were not statistically significant. Importantly, the regression slope between microbial C:N and substrate C:N was close to zero (slope = 0.04), yielding a homeostasis index H of 25. This indicates extremely strong stoichiometric homeostasis, suggesting that microbial elemental composition was largely insensitive to changes in external resource stoichiometry, reflecting pronounced physiological regulation.

### 3.6. Association Networks Between Multicomponent Soil Stoichiometry and Microbial Functional Attributes

**Figure 5:**
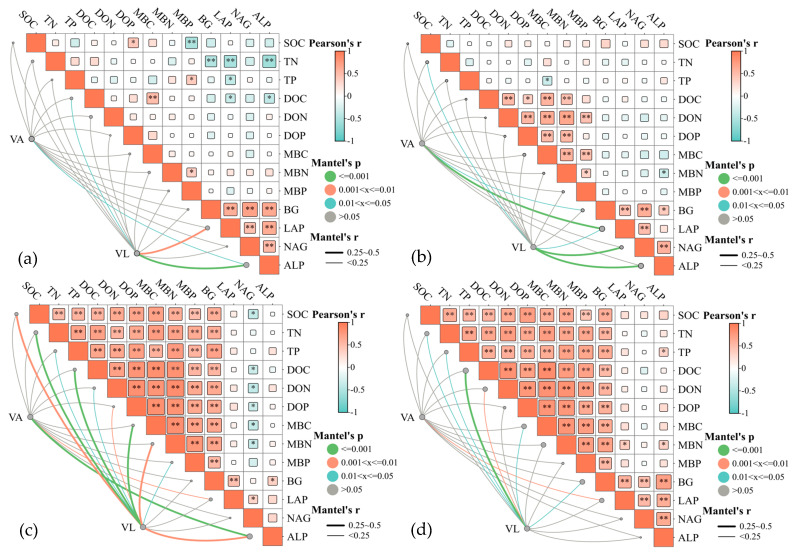
Mantel correlation heatmap between soil total nutrients, soluble nutrients, microbial biomass, extracellular enzyme activity, and vector characteristics. (**a**) Rhizosphere and bulk soils under CF treatment, (**b**) rhizosphere and bulk soils under OIF treatment, (**c**) bulk soils under CF and OIF treatments, and (**d**) rhizosphere soils under CF and OIF treatments. Line thickness indicates the strength of the correlation, and different colors represent various levels of statistical significance. * indicates statistical significance (* *p* < 0.05; ** *p* < 0.01).

Correlation analyses (Figure 5) showed that OIF strengthened the correlations among soluble C, N, and P pools, as well as among microbial biomass C, N, and P pools. However, OIF reduced the correlations between SOC, TN, TP and extracellular enzyme activities (Figure 5a,b). In bulk soils, NAG activity was significantly negatively correlated with SOC, soluble C-N-P nutrients, and microbial biomass C and N (Figure 5c). This pattern was not observed in rhizosphere soils. Instead, rhizosphere soils showed markedly strengthened correlations among extracellular enzyme activities (Figure 5d). Mantel test results indicated that the relationships between vector length/angle and soil chemical properties did not differ substantially between OIF and CF. In bulk soils, vector length showed stronger and significant correlations with soil stoichiometry, microbial biomass stoichiometry, and extracellular enzyme stoichiometry.

### 3.7. Key Pathway Analysis of Drivers Regulating Microbial Element Use Efficiency

**Figure 6:**
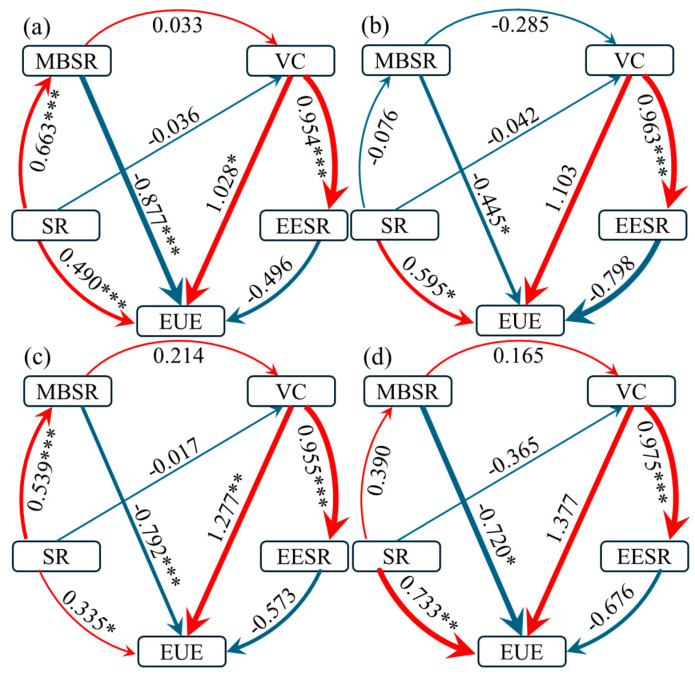
PLS-PM of the relationships between stoichiometric ratios, microbial biomass stoichiometric ratios, extracellular enzyme stoichiometric ratios, and vector characteristics on microbial element use efficiency. (**a**) Influence path of element use efficiency under CF treatment, (**b**) influence path of element use efficiency under OIF treatment, (**c**) influence path of non-rhizosphere element use efficiency under CF and OIF treatments, and (**d**) influence path of rhizosphere element use efficiency under CF and OIF treatments. SR: stoichiometric ratio (CN, CP, and NP); MBSR: microbial biomass stoichiometric ratio (MBC:MBN, MBC:MBP, and MBN:MBP); VC: vector characteristics (VL and VA); EESR: extracellular enzyme stoichiometric ratio (BG:(NAG + LAP), BG:ALP, and (NAG + LAP):ALP); EUE: element use efficiency (CUE, NUE, and PUE). Red and blue arrows represent positive and negative correlations, respectively. The numbers indicate path coefficients, and the arrow width represents the strength of the correlation, with wider arrows indicating stronger correlations. * *p* < 0.05, ** *p* < 0.01, and *** *p* < 0.001.

The PLS-PM results (Figure 6) indicated that microbial EUE in rhizosphere soils was primarily driven by soil stoichiometric ratios and microbial biomass stoichiometric ratios (Table S1; Figure 6d), and in bulk soils, it was jointly and significantly influenced by soil stoichiometric ratios, microbial biomass stoichiometric ratios, and vector characteristics (Table S2; Figure 6c). In addition, in both bulk soils and CF-managed soils, soil stoichiometric ratios significantly regulated microbial biomass stoichiometric ratios (Figure 6a,c). Under OIF, microbial EUE was mainly determined by soil stoichiometric ratios and microbial biomass stoichiometric ratios (Figure 6b), whereas under CF, it was also significantly influenced by vector characteristics (Figure 6a). Notably, organic–inorganic compound fertilizer altered the direction of key regulatory pathways. Specifically, the positive effects of soil stoichiometric ratios on microbial biomass stoichiometric ratios, and of microbial biomass stoichiometric ratios on vector characteristics, shifted to negative effects. In both rhizosphere and bulk soils, extracellular enzyme stoichiometric ratios were significantly and positively regulated by vector characteristics. Meanwhile, OIF weakened the influence of microbial biomass stoichiometry on EUE but strengthened the contribution of extracellular enzyme stoichiometry to EUE.

## 4. Discussion

### 4.1. Seasonal Dynamics of Soil Nutrients and Extracellular Enzyme Activity

The results of this study indicate that the chemical properties of soil and microbial biomass in walnut plantations exhibit significant seasonal fluctuations, although the changes in different indicators are not synchronized (Table 1). TN and TP showed a trend of decreasing and then increasing, with no significant seasonal differences, while DOC, DN, and microbial biomass (MBC, MBN, and MBP) showed more sensitive seasonal responses. This finding emphasizes the typical characteristics of alpine ecosystems, where total nutrients are less likely to show significant changes over short seasonal scales, while dissolved nutrients and microbial biomass, as more rapidly cycling pools, can respond more quickly to changes in temperature, moisture, and vegetation growth cycles [34,35]. During the growing season in July, DOC decreased significantly both in the rhizosphere and non-rhizosphere, while microbial biomass (MBC, MBN, and MBP) exhibited a stage-wise decline under conventional fertilization treatment. This was likely due to the rapid consumption of available substrates by microbes and the competition for nutrients between plants and microbes [36]. In particular, in the rhizosphere, the intense absorption of inorganic nutrients by plant roots during the growing season may enhance microbial absorption of dissolved nutrients, thus accelerating DOC turnover and causing a short-term decline in microbial biomass [37]. In contrast, MBC significantly decreased in July but rebounded in November under the combined organic and inorganic fertilization treatment. This dynamic change suggests that while exogenous nutrient inputs suppressed microbial biomass accumulation during the growing season due to intensified competition, they may instead promote biomass recovery and compensatory growth in the later stages of the growing season by improving substrate quality or alleviating resource limitations [38,39]. This nonlinear response reflects the seasonal characteristics of fertilization effects in alpine environments and indicates that microbial communities possess a certain degree of plasticity and adaptability [40,41]. The activities of BG, LAP, NAG, and ALP peaked in July, indicating that soil microbes had a stronger potential for substrate acquisition and nutrient mineralization during the growing season [42,43]. Notably, the application of organic–inorganic compound fertilizer significantly increased BG and LAP activities in both the rhizosphere and non-rhizosphere in July and made the seasonal peaks of NAG and ALP in the rhizosphere more pronounced (Table 2). This suggests that fertilization not only altered resource availability but also encouraged microbes to adopt a more “investment-driven” strategy, increasing extracellular enzyme synthesis during the period of greatest resource competition (July) to accelerate the acquisition of key nutrients such as N and P [44]. This phenomenon is also consistent with the subsequent vector model results (Table 2; Figure 1), which showed that microbial metabolic limitations were driven by the joint constraints of C-N and C-P ratios. Microbes need to simultaneously enhance their capacity to acquire both N and P to maintain metabolic balance.

### 4.2. Combined Organic and Inorganic Fertilization and Rhizosphere Effects Synergistically Enhance Microbial Stoichiometric Homeostasis

This study found that combined organic and inorganic fertilization significantly enhanced the internal stoichiometric balance of microbial biomass C:N:P and soil resources C:N:P, especially in the rhizosphere soil. In this region, the C:N ratio of microbial biomass was nearly unaffected by changes in substrate C:N, showing extreme stoichiometric homeostasis (Figure 4). This finding supports our hypothesis (1) that combined organic and inorganic fertilization alleviates direct resource limitations for microbial growth by providing available nutrients, allowing microbes to maintain a more stable internal chemical composition, which is a key physiological strategy for coping with environmental fluctuations [45,46]. However, we also observed that combined organic and inorganic fertilization did not consistently increase CUE and, in fact, showed a lower CUE trend during the growing season (July) (Figure 3). This contradicts previous research findings [47,48] and our hypothesis, revealing the complexity of nutrient management in alpine sandy ecosystems. This discrepancy is mainly attributed to the metabolic trade-offs induced by combined organic and inorganic fertilization. Fertilization (especially N fertilization) alleviates C limitation while rapidly intensifying microbial competition for P, which results in a worsening of the C:P imbalance and a significant increase in ALP activity in the rhizosphere under combined organic and inorganic fertilization (Figure 2; Table 2). To acquire the limited P, microbes must allocate more energy and C substrates to the synthesis and secretion of phosphatases, which increases the metabolic cost and reduces CUE [17,18]. From the microbial community perspective (Figure S1), in the rhizosphere soil under combined organic and inorganic fertilization, the relative abundance of Actinobacteria was significantly higher than in the conventional fertilization treatment, as this group is generally more capable of organic phosphorus mineralization [49]. Additionally, metagenomic analysis (Figure S2) showed that genes related to organic phosphorus mineralization were more abundant in the microbial communities under combined organic and inorganic fertilization, and this indicates that combined organic and inorganic fertilization not only intensifies P limitation but also enriches microbial groups with a competitive advantage for P acquisition. This adaptive shift in community functional traits may explain how microbial communities maintain internal homeostasis in a nutrient-imbalanced environment [50]. Our PLS-PM analysis clearly supports this mechanism (Figure 6). Organic–inorganic fertilization altered the regulatory path of microbial biomass stoichiometry on vector characteristics from positive to negative (Figure 6b), suggesting that larger microbial biomass is associated with stronger nutrient limitation [46]. Furthermore, the regulatory path of extracellular enzyme activity on EUE was enhanced, indicating that in the fertilized system, the key factor determining microbial growth efficiency was not how much biomass they had, but the metabolic cost of acquiring resources [51,52]. Therefore, in the P-deficient alpine sandy system, this fertilization method, while enhancing microbial physiological stability, may trigger a “C-P metabolic trade-off”, shifting microbes from a “growth efficiency-first” strategy to a “nutrient acquisition-first” strategy, ultimately limiting the potential for soil C sequestration [28,53].

### 4.3. Seasonal Discrepancies in Microbial Element Use Efficiency Reflect Strategic Adaptations

The results of this study also support hypothesis (2), showing that microbial EUE exhibits significant seasonal differentiation (Figure 3). CUE peaks in July under optimal water and temperature conditions, which corresponds to higher substrate availability (DOC and DON) and microbial activity (BG and LAP). During this period, microbes tend to invest resources in rapid growth, leading to higher CUE (Table 1 and Table 2, and Figure 3a). In contrast, in late plant growth and dormancy phases (November and March), nitrogen NUE and PUE significantly increase (Figure 3b,c). This likely represents an adaptive survival strategy: under low temperatures and freeze–thaw stress, microbial growth rates are suppressed, and microbes redirect limited N and P resources into biomass, potentially synthesizing more storage or protective compounds to withstand environmental pressures [21,54]. These seasonal variations in CUE, NUE, and PUE highlight the resource allocation trade-offs microbes face when responding to periodic environmental stresses. In late autumn (October), the relative abundance of oligotrophic bacterial groups, such as Actinobacteria and Acidobacteria, was significantly higher in bulk soil compared with the rhizosphere (Figure S1). These groups are typically associated with lower metabolic rates and higher nutrient conservation efficiency [55,56]. Notably, it was during this period (November) that we observed the peak in microbial NUE and PUE (Figure 3b,c). Therefore, we suppose that the oligotrophic-dominant community structure in the soil during autumn and winter may be one of the driving factors behind the high nutrient use efficiency. More importantly, the rhizosphere and bulk soils exhibited different regulatory pathways (Figure 6). In the rhizosphere, EUE was primarily regulated by soil stoichiometry and microbial biomass, whereas in the non-rhizosphere, vector characteristics became an additional significant driver. This suggests that in the rhizosphere, where nutrient input is active and root exudates are abundant, microbial metabolic efficiency is more directly dependent on the immediate availability of nutrients. In contrast, in the non-rhizosphere, microbial metabolic efficiency is more strongly determined by the intensity and type of nutrient limitations they perceive [57,58]. Further analysis of microbial β-diversity confirmed that the microbial communities in the rhizosphere and bulk soils exhibited distinct structures (Figure S3). The rhizosphere was enriched with more members of the Pseudomonadota, which respond rapidly to easily available C sources (Figure S1), explaining why rhizosphere EUE is more directly driven by immediate resource availability [59,60]. Meanwhile, the non-rhizosphere community structure is more stable, and its metabolic activity is more regulated by resource scarcity (vector characteristics) [61]. This microhabitat differentiation demonstrates the metabolic specificity of the rhizosphere and highlights the importance of considering microhabitat scale when assessing the effects of fertilization management strategies.

### 4.4. Environmental Stress Drives Seasonal Dynamics of Microbial Nutrient Limitation

The vector model results in this study show that microbial growth is jointly limited by C-N and C-P throughout the year (Figure 1), consistent with the impoverished soil background of alpine sandy ecosystems [62]. However, the intensity of limitation exhibits clear seasonal dynamics, with the vector length under conventional fertilization treatment being significantly higher in winter and spring compared with summer. This suggests that freeze–thaw cycles and water stress are key abiotic drivers intensifying microbial nutrient limitations. The freeze–thaw process disrupts microbial cells and alters soil aggregation, potentially releasing large amounts of available C, but at the same time, it exacerbates the risk of nutrient fixation or leaching, leading to stoichiometric imbalance [8,19]. This environment-driven change in the intensity of nutrient limitations provides insights into the seasonal patterns of EUE. During the most intense limitation in winter and spring, microbes show the highest nutrient conservation efficiency (NUE and PUE), while during the growing season when limitations are alleviated, they shift towards maximizing growth efficiency (CUE).

### 4.5. Study Limitations and Future Directions

This study is the first to systematically elucidate the synergistic changes and regulatory mechanisms of microbial C-N-P use efficiency under fertilization management in the alpine sandy artificial forest ecosystem. It reveals that microbial EUE does not change independently but is governed by a synergistic response system involving environmental stress, elemental stoichiometry, and microbial physiological strategies on a seasonal scale. The decoupling of regulatory pathways between the rhizosphere and non-rhizosphere indicates the importance of soil heterogeneity in predicting ecosystem functions. This study also highlights the need for careful consideration of nitrogen-induced phosphorus limitation in current nitrogen-based afforestation management in the alpine sandy land, as it may negatively impact soil carbon sequestration. Future restoration practices should incorporate nitrogen–phosphorus co-application or microbial inoculants to enhance phosphorus activation, balancing microbial metabolic trade-offs and improving both ecosystem productivity and carbon sequestration capacity.

This study also has several limitations. First, the seasonal changes in microbial community composition were not addressed, although community succession may be an inherent factor driving changes in EUE [27]. Future studies should combine seasonal dynamic sampling with metagenomic and transcriptomic techniques to track how the expression of functional genes in key microbial groups directly responds to fertilization and seasonal changes, thus providing a comprehensive analysis of the pathway from community succession to changes in EUE. Second, the lack of data on seasonal changes in plant root physiological activities (such as exudate composition) limits our understanding of the mechanisms driving rhizosphere effects. Future research could integrate molecular biology techniques with stable isotope labeling to trace the flow and transformation efficiency of carbon, nitrogen, and phosphorus in the plant–soil–microbe system. Additionally, gradient fertilization experiments should be conducted to precisely identify threshold effects in nutrient management, thereby providing scientific insights for adaptive management in vulnerable alpine ecosystems [63,64,65].

## 5. Conclusions

This study demonstrates that while integrated organic–inorganic fertilization (OIF) enhances microbial stoichiometric homeostasis, it triggers a seasonal metabolic trade-off that limits carbon use efficiency (CUE) during the peak growing season. Microbial EUE exhibited strategic seasonal differentiation, with microbes prioritizing growth during the summer and shifting to efficient nutrient conservation in winter. A decoupling of EUE between the rhizosphere and non-rhizosphere domains was observed, highlighting the importance of soil heterogeneity in predicting ecosystem functions. Therefore, future adaptive management should prioritize stoichiometrically balanced fertilization by lowering N:P input ratios to alleviate phosphorus limitations. Furthermore, the functional differences between rhizosphere and non-rhizosphere microhabitats should be fully considered. This approach will help to promote both ecosystem productivity and the long-term retention of soil nitrogen and phosphorus. Despite these insights, this study is limited by the lack of a no-fertilizer control and long-term community succession data.

## Figures and Tables

**Table 1:** Soil total nutrients, soluble nutrients, and microbial biomass under different treatments.

Indicator	Treatment	March	July	November
SOC	CF-R	6.30 ± 0.24 Aa	5.73 ± 0.47 Aa	5.98 ± 0.62 Aa
(g·kg^−1^)	CF-B	5.57 ± 0.23 Aa	5.23 ± 0.45 Aa	5.51 ± 0.28 Aa
	OIF-R	8.01 ± 0.56 Aa	8.21 ± 0.51 Aa	8.50 ± 0.36 Aa
	OIF-B	7.25 ± 0.37 Aa	7.39 ± 0.68 Aa	7.62 ± 0.50 Aa
TN	CF-R	0.767 ± 0.011 Aa	0.649 ± 0.034 Bb	0.762 ± 0.020 ABa
(g·kg^−1^)	CF-B	0.771 ± 0.024 Aa	0.652 ± 0.042 Ab	0.732 ± 0.019 Aab
	OIF-R	0.903 ± 0.018 Aa	0.887 ± 0.026 Aa	0.891 ± 0.021 Aa
	OIF-B	0.888 ± 0.032 Aa	0.879 ± 0.010 Aa	0.883 ± 0.022 Aa
TP	CF-R	0.379 ± 0.032 Aa	0.344 ± 0.034 Aa	0.384 ± 0.019 Aa
(g·kg^−1^)	CF-B	0.364 ± 0.026 Aa	0.358 ± 0.040 Aa	0.401 ± 0.026 Aa
	OIF-R	0.559 ± 0.052 Aa	0.549 ± 0.036 Aa	0.531 ± 0.018 Aa
	OIF-B	0.531 ± 0.015 Aa	0.554 ± 0.020 Aa	0.547 ± 0.031 Aa
DOC	CF-R	121.6 ± 5.74 Aa	90.56 ± 11.38 Ab	84.43 ± 5.33 Ab
(mg·kg^−1^)	CF-B	118.1 ± 4.20 Aa	78.19 ± 6.30 Bb	82.84 ± 8.61 Bb
	OIF-R	197.6 ± 6.9 ABa	152.6 ± 12.0 Bb	201.5 ± 8.7 Aa
	OIF-B	170.1 ± 9.8 Aa	134.1 ± 9.5 Ab	161.7 ± 4.5 Aab
DN	CF-R	24.61 ± 2.78 Aa	20.93 ± 2.40 Aa	18.56 ± 0.79 Aa
(mg·kg^−1^)	CF-B	24.88 ± 1.32 Aa	26.14 ± 2.36 Aa	17.91 ± 1.40 Ab
	OIF-R	53.81 ± 2.57 Aa	33.96 ± 2.33 Bb	58.31 ± 0.74 Aa
	OIF-B	42.29 ± 4.10 Aa	35.77 ± 1.61 Aa	44.74 ± 2.53 Aa
DP	CF-R	2.39 ± 0.43 Aa	1.93 ± 0.28 Aa	1.88 ± 0.27 Aa
(mg·kg^−1^)	CF-B	1.97 ± 0.24 Aa	1.74 ± 0.24 Aa	1.47 ± 0.18 Aa
	OIF-R	5.27 ± 0.49 ABab	3.79 ± 0.50 Bb	6.65 ± 0.36 Aa
	OIF-B	5.10 ± 0.58 Aa	4.27 ± 0.20 Aa	5.25 ± 0.37 Aa
MBC	CF-R	82.49 ± 5.46 Aa	73.01 ± 3.78 ABa	51.62 ± 4.09 Bb
(mg·kg^−1^)	CF-B	69.67 ± 6.57 Aa	58.80 ± 3.65 Aab	41.72 ± 7.35 Ab
	OIF-R	146.9 ± 9.1 ABa	115.6 ± 2.6 Bb	163.4 ± 9.5 Aa
	OIF-B	136.8 ± 4.3 Aa	89.5 ± 3.0 Bb	139.1 ± 5.6 Aa
MBN	CF-R	25.77 ± 1.59 Aa	29.66 ± 1.41 Aa	24.50 ± 3.46 Aa
(mg·kg^−1^)	CF-B	26.18 ± 1.20 Aa	24.54 ± 1.29 Aa	23.85 ± 1.00 Aa
	OIF-R	49.57 ± 4.22 ABab	39.54 ± 2.81 Bb	58.40 ± 2.90 Aa
	OIF-B	47.68 ± 1.84 Ab	36.68 ± 1.58 Bc	55.89 ± 1.99 Aa
MBP	CF-R	8.90 ± 0.50 Aa	7.73 ± 0.93 Aa	8.15 ± 1.62 Aa
(mg·kg^−1^)	CF-B	8.64 ± 1.31 Aa	7.66 ± 1.28 Aa	7.76 ± 1.09 Aa
	OIF-R	14.06 ± 1.66 Aa	13.12 ± 0.86 Aa	16.43 ± 1.23 Aa
	OIF-B	11.75 ± 0.66 Aab	11.07 ± 1.10 Ab	14.45 ± 0.56 Aa

SOC: soil organic carbon; TN: total nitrogen; TP: total phosphorus; DOC: dissolved organic carbon; DN: dissolved nitrogen; DP: dissolved phosphorus; MBC: microbial biomass carbon; MBN: microbial biomass nitrogen; MBP: microbial biomass phosphorus. CF-R: the rhizosphere under CF; CF-B: the non-rhizosphere under CF; OIF-R: the rhizosphere under OIF; OIF-B: the non-rhizosphere under OIF. Different lowercase letters (a, b) indicate significant differences between months within the same treatment at *p* < 0.05; different uppercase letters (A, B) indicate significant differences at *p* < 0.01.

**Table 2:** Extracellular enzyme activity under different treatments.

Indicator	Treatment	March	July	November
BG	CF-R	0.517 ± 0.053 Bb	0.963 ± 0.058 Aa	0.705 ± 0.056 ABb
(μmol·g^−1^·h^−1^)	CF-B	0.542 ± 0.048 Ab	0.716 ± 0.022 Aa	0.633 ± 0.048 Aab
	OIF-R	1.082 ± 0.051 Ab	1.279 ± 0.078 Aa	1.247 ± 0.051 Aa
	OIF-B	0.922 ± 0.067 Ab	1.173 ± 0.041 Aa	1.087 ± 0.067 Aab
LAP	CF-R	0.165 ± 0.019 Bb	0.405 ± 0.036 Aa	0.240 ± 0.023 Bb
(μmol·g^−1^·h^−1^)	CF-B	0.146 ± 0.027 Bb	0.314 ± 0.045 Aa	0.187 ± 0.027 ABb
	OIF-R	0.269 ± 0.019 Ab	0.406 ± 0.029 Aa	0.365 ± 0.038 Aab
	OIF-B	0.190 ± 0.025 Ab	0.299 ± 0.043 Aa	0.271 ± 0.008 Aab
NAG	CF-R	0.061 ± 0.007 Bc	0.187 ± 0.012 Aa	0.147 ± 0.009 Ab
(μmol·g^−1^·h^−1^)	CF-B	0.106 ± 0.025 Aa	0.155 ± 0.021 Aa	0.112 ± 0.018 Aa
	OIF-R	0.087 ± 0.013 Bb	0.199 ± 0.027 Aa	0.161 ± 0.021 ABab
	OIF-B	0.068 ± 0.012 Bb	0.144 ± 0.017 Aa	0.094 ± 0.011 ABab
ALP	CF-R	0.344 ± 0.032 Bb	0.699 ± 0.039 Aa	0.414 ± 0.026 Bb
(μmol·g^−1^·h^−1^)	CF-B	0.311 ± 0.021 Bb	0.623 ± 0.014 Aa	0.335 ± 0.050 Bb
	OIF-R	0.561 ± 0.044 Bb	0.782 ± 0.053 Aa	0.574 ± 0.025 Bb
	OIF-B	0.475 ± 0.043 Aa	0.536 ± 0.039 Aa	0.388 ± 0.039 Aa

BG: β-glucosidase, C-acquiring enzyme; LAP: leucine aminopeptidase, N-acquiring enzyme; NAG: β-N-acetylglucosaminidase, N-acquiring enzyme; ALP: alkaline phosphatase, P-acquiring enzyme. CF-R: the rhizosphere under CF; CF-B: the non-rhizosphere under CF; OIF-R: the rhizosphere under OIF; OIF-B: the non-rhizosphere under OIF. Different lowercase letters (a, b) indicate significant differences between months within the same treatment at *p* < 0.05; different uppercase letters (A, B) indicate significant differences at *p* < 0.01.

## Data Availability

The data presented in this study are available on request from the corresponding author.

## Supplementary Materials

The following supporting information can be downloaded at: https://www.mdpi.com/article/10.3390/microorganisms14061186/s1, Figure S1: Phylum-level microbial community composition in rhizosphere and bulk soils under combined organic and inorganic fertilization and conventional organic fertilization treatments; Figure S2: Changes in functional genes related to organic phosphorus mineralization in rhizosphere and bulk soils under combined organic and inorganic fertilization; Figure S3: Beta diversity analysis of microbial communities under different treatments. Table S1: Soil stoichiometric ratios and microbial biomass stoichiometric ratios under different treatments; Table S2: Extracellular enzyme stoichiometric ratios under different treatments.
