# Supplementary material for: Integrated Organic–Inorganic Fertilization Enhances Microbial Stoichiometric Homeostasis but Triggers Seasonal Metabolic Trade-Offs in an Alpine Sandy Ecosystem
Source: Microorganisms. 2026 May 25;14(6):1186. doi: 10.3390/microorganisms14061186 (PMC13303656; doi:10.3390/microorganisms14061186)
Supplement: Supplementary file 1 [file microorganisms-14-01186-s001.zip › microorganisms-4336245-supplementary.pdf]

Table S1 Soil stoichiometric ratios and microbial biomass stoichiometric ratios under different treatments.

Indicators	Treatment	March	July	November
SOC:TN	CF-R	8.22±0.30Aa	8.90±0.80Aa	7.90±0.87Aa
	CF-B	7.29±0.45Aa	8.14±0.77Aa	7.54±0.40Aa
	OIF-R	8.93±0.76Aa	9.30±0.66Aa	9.53±0.25Aa
	OIF-B	8.28±0.74Aa	8.43±0.82Aa	8.66±0.63Aa
SOC:TP	CF-R	17.25±1.63Aa	17.70±2.66Aa	15.76±1.74Aa
	CF-B	15.87±1.65Aa	16.57±3.78Aa	14.15±1.44Aa
	OIF-R	15.23±2.09Aa	15.20±1.11Aa	16.07±0.74Aa
	OIF-B	13.67±0.60Aa	13.47±1.45Aa	14.05±0.99Aa
TN:TP	CF-R	2.09±0.16Aa	1.95±0.15Aa	2.02±0.15Aa
	CF-B	2.17±0.18Aa	1.95±0.28Aa	1.86±0.13Aa
	OIF-R	1.70±0.18Aa	1.66±0.14Aa	1.69±0.07Aa
	OIF-B	1.69±0.10Aa	1.60±0.07Aa	1.64±0.11Aa
DOC:DN	CF-R	5.25±0.58Aa	4.68±0.76Aa	4.55±0.22Aa
	CF-B	4.79±0.25Aab	3.15±0.44Ab	4.92±0.82Aa
	OIF-R	3.69±0.15Aab	4.65±0.56Aa	3.46±0.18Ab
	OIF-B	4.28±0.56Aa	3.76±0.24Aa	3.69±0.28Aa
DOC:DP	CF-R	61.58±12.74Aa	44.09±6.36Aa	49.39±6.49Aa
	CF-B	63.25±5.91Aa	47.47±4.68Aa	61.05±9.21Aa
	OIF-R	39.45±4.32Aa	46.00±9.36Aa	30.68±1.93Aa
	OIF-B	35.30±3.92Aa	31.54±2.07Aa	31.60±2.33Aa
DN:DP	CF-R	11.42±1.44Aa	10.05±1.3Aa	11.25±2.13Aa
	CF-B	13.45±1.56Aa	16.22±2.36Aa	12.90±1.55Aa
	OIF-R	10.74±1.26Aa	9.67±1.26Aa	8.90±0.50Aa
	OIF-B	9.07±1.77Aa	8.41±0.33Aa	8.59±0.31Aa
MBC:MBN	CF-R	3.31±0.37Aa	2.48±0.16Ab	2.24±0.21Ab
	CF-B	2.68±0.27Aa	2.47±0.30Aab	1.71±0.26Ab
	OIF-R	3.08±0.32Aa	3.00±0.22Aa	2.87±0.31Aa
	OIF-B	2.90±0.19Aa	2.45±0.10Aa	2.51±0.16Aa
MBC:MBP	CF-R	9.48±0.95Aa	9.91±0.88Aa	7.44±1.26Aa
	CF-B	8.75±1.04Aa	9.81±2.90Aa	5.50±0.83Aa
	OIF-R	11.34±1.70Aa	8.95±0.43Aa	10.13±0.70Aa
	OIF-B	11.78±0.57Aa	8.45±0.79Bb	9.69±0.50Abb
MBN:MBP	CF-R	2.96±0.26Aa	4.07±0.42Aa	3.35±0.49Aa
	CF-B	3.59±0.81Aa	3.82±0.76Aa	3.30±0.33Aa
	OIF-R	3.61±0.22Aa	3.11±0.37Aa	3.67±0.36Aa
	OIF-B	4.15±0.36Aa	3.47±0.34Aa	3.91±0.27Aa

SOC: Soil organic carbon; TN: Total nitrogen; TP: Total phosphorus; DOC: Dissolved organic carbon; DN: Dissolved nitrogen; DP: Dissolved phosphorus; MBC: Microbial biomass carbon; MBN：Microbial biomass nitrogen; MBP: Microbial biomass phosphorus. CF-R: The rhizosphere under CF; CF-B: The non-rhizosphere under CF; OIF-R: The rhizosphere under OIF; OIF-B: The non-rhizosphere under OIF. The different letters on the same line indicate significant differences between the same treatment in different months (lowercase letters: $P < 0.05$, uppercase letters: $P < 0.01$).

Table S2 Extracellular enzyme stoichiometric ratios under different treatments.

Indicators	Treatment	March	July	November
BG/(LAP+NAG)	CF-R	2.36±0.3Aa	1.66±0.16Ab	1.86±0.21Aab
	CF -B	2.33±0.23Aa	1.58±0.15Ab	2.29±0.29Aa
	OIF-R	3.09±0.24Aa	2.13±0.07Bb	2.42±0.22ABb
	OIF-B	3.85±0.49Aa	2.8±0.29Aa	3.03±0.28Aa
BG/ALP	CF-R	1.54±0.15Aa	1.39±0.1Aa	1.71±0.11Aa
	CF -B	1.75±0.13ABab	1.15±0.03Bb	2.16±0.38Aa
	OIF-R	1.97±0.13ABb	1.64±0.07Bb	2.19±0.11Aa
	OIF-B	2.09±0.24Ab	2.24±0.14Ab	2.91±0.26Aa
(LAP+NAG)/ALP	CF-R	0.73±0.16Aa	0.87±0.08Aa	0.96±0.11Aa
	CF -B	0.8±0.11Aa	0.76±0.07Aa	1.03±0.22Aa
	OIF-R	0.66±0.06Ab	0.78±0.06Aab	0.93±0.07Aa
	OIF-B	0.56±0.05Bb	0.84±0.1ABab	0.99±0.12Aa

BG: β-glucosidase, C-acquiring enzyme; LAP: leucine aminopeptidase, N-acquiring enzyme; NAG: β-N-acetylglucosaminidase, N- acquiring enzyme; ALP: alkaline phosphatase, P- acquiring enzyme. CF-R: The rhizosphere under CF; CF-B: The non-rhizosphere under CF; OIF-R: The rhizosphere under OIF; OIF-B: The non-rhizosphere under OIF. The different letters on the same line indicate significant differences between the same treatment in different months (lowercase letters: $P < 0.05$, uppercase letters: $P < 0.01$).

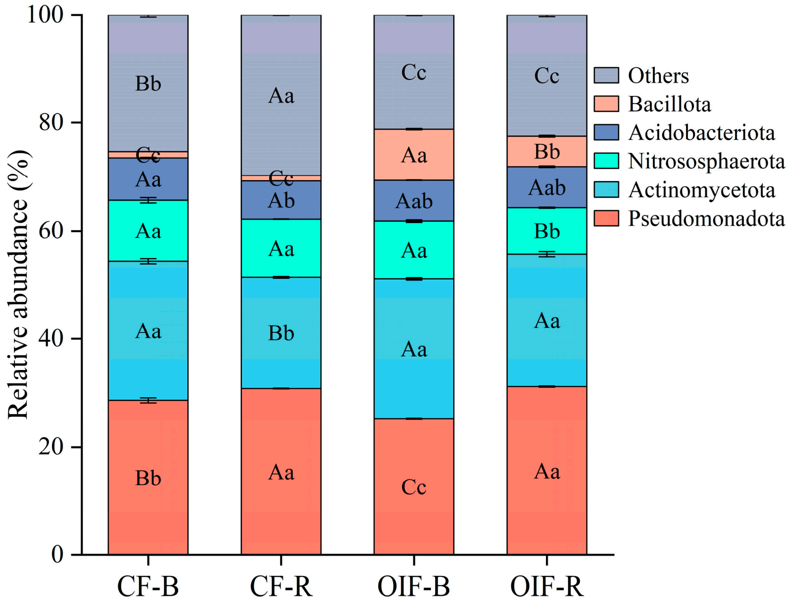

Fig. S1 Phylum-level microbial community composition in rhizosphere and bulk soils under combined organic and inorganic fertilization and conventional organic fertilization treatments. Only the top five taxa with the highest relative abundance among classified groups are shown; the remaining taxa are grouped as “Others” . Different uppercase letters ($P < 0.01$) and lowercase letters ($P < 0.05$) indicate significant differences between treatments.

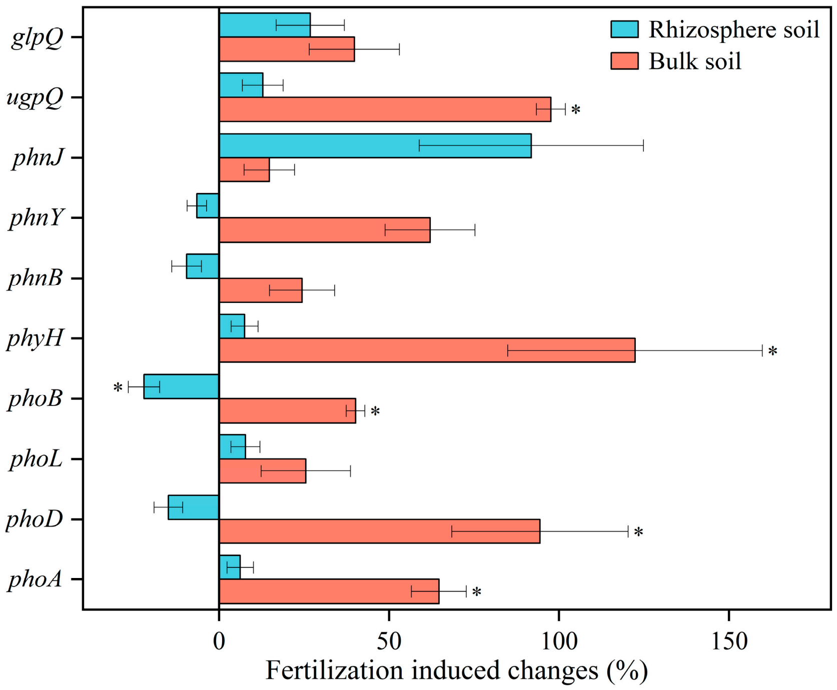

Fig. S2 Changes in functional genes related to organic phosphorus mineralization in rhizosphere and bulk soils under combined organic and inorganic fertilization (mean ± SE). Alkaline phosphatases: *phoA, phoD, phoL, phoB*; Phytase: *phyH*; C-P lyase system: *phnB, phnY, phnJ*; Phosphate monoester/diester hydrolysis: *ugpQ, glpQ*. * indicate statistical significance ($P < 0.05$).

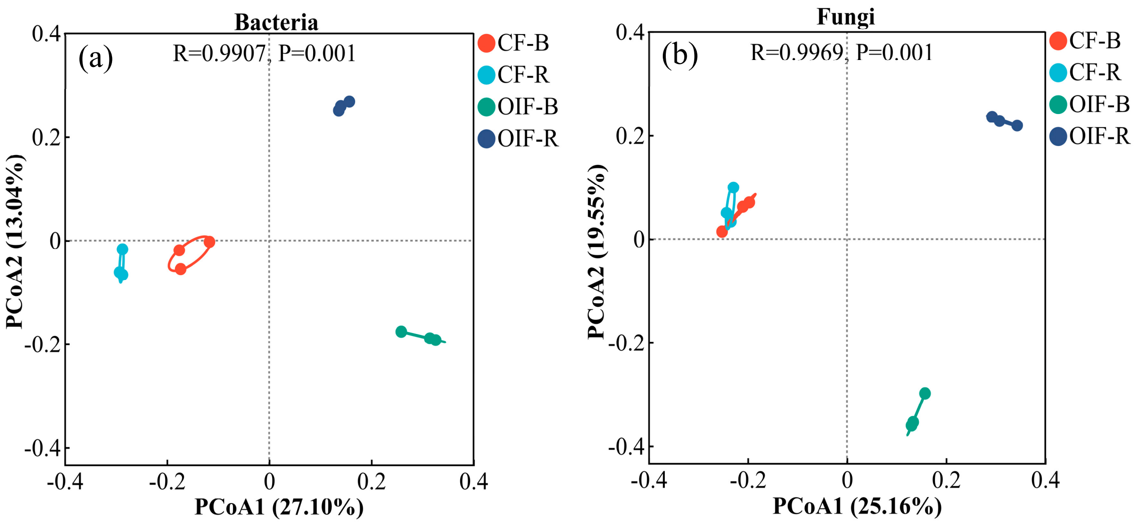

Fig. S3 Beta diversity analysis of microbial communities under different treatments. (a) Bacterial community (PCoA), (b) Fungal community (PCoA).
